# Intraspecific variation in vertical habitat use by tiger sharks (*Galeocerdo cuvier*) in the western North Atlantic

**DOI:** 10.1002/ece3.1053

**Published:** 2014-04-12

**Authors:** Jeremy J Vaudo, Bradley M Wetherbee, Guy Harvey, Richard S Nemeth, Choy Aming, Neil Burnie, Lucy A Howey-Jordan, Mahmood S Shivji

**Affiliations:** 1 The Guy Harvey Research Institute, Nova Southeastern University Oceanographic CenterDania Beach, Florida; 2 Department of Biological Sciences, University of Rhode IslandKingston, Rhode Island; 3 Center for Marine and Environmental Studies, University of the Virgin IslandsCharlotte Amalie, St. Thomas, US Virgin Islands; 4 The Bermuda Shark ProjectFlatts, Bermuda; 5 Microwave Telemetry, Inc.Columbia, Maryland

**Keywords:** Depth distribution, dive behavior, individual variation, movements, Pop-up Archival Transmitting tag, telemetry

## Abstract

Tiger sharks (*Galeocerdo cuvier*) are a wide ranging, potentially keystone predator species that display a variety of horizontal movement patterns, making use of coastal and pelagic waters. Far less, however, is known about their vertical movements and use of the water column. We used pop-up satellite archival tags with two data sampling rates (high rate and standard rate tags) to investigate the vertical habitat use and diving behavior of tiger sharks tagged on the Puerto Rico–Virgin Islands platform and off Bermuda between 2008 and 2009. Useable data were received from nine of 14 sharks tagged, tracked over a total of 529 days. Sharks spent the majority of their time making yo-yo dives within the upper 50 m of the water column and considerable time within the upper 5 m of the water column. As a result, sharks typically occupied a narrow daily temperature range (∼2°C). Dives to greater than 200 m were common, and all sharks made dives to at least 250 m, with one shark reaching a depth of 828 m. Despite some similarities among individuals, a great deal of intraspecific variability in vertical habit use was observed. Four distinct depth distributions that were not related to tagging location, horizontal movements, sex, or size were detected. In addition, similar depth distributions did not necessitate similar dive patterns among sharks. Recognition of intraspecific variability in habitat use of top predators can be crucial for effective management of these species and for understanding their influence on ecosystem dynamics.

## Introduction

Marine ecosystems by virtue of their three dimensional habitat allow evolution of a complex interaction of horizontal and vertical movements by highly mobile species, including large apex predators. Elucidating the movements of large apex predators is a key element of understanding ecosystem dynamics for a number of reasons, including defining the areas and scales at which predators exert top-down pressure through consumptive and risk effects, and determining the level of connectivity between ecosystems. Many shark species are large mobile predators that occupy upper trophic levels (Cortés 1999), and as such their movements are of great interest for the above reasons as well as for understanding their interactions with fisheries and making informed management efforts. Furthermore, because many shark species are experiencing worldwide declines (e.g., Musick et al. 1993; Baum et al. 2003; Ferretti et al. 2010) and there are urgent concerns about their population statuses, examining the movements of sharks to gain insight into their migration pathways, population structure, spatial vulnerability to fisheries and ecological impacts has taken on increased importance in recent years to enhance conservation efforts (Sims 2010).

Indeed, sharks have been the focus of many movement studies using a variety of telemetry technologies. Most of these studies, however, have focused on horizontal movements of sharks, with far fewer studies on vertical movements (Speed et al. 2010), even though understanding movements in the vertical dimension is just as important to understanding their overall spatial behavior. Knowledge of vertical movements provides insight into how animals use their environment (i.e., the water column), how animals with similar geographic distributions partition habitats (e.g., Musyl et al. 2011), and the potential for species interactions in the vertical dimension, which can effect trophic dynamics (e.g., trophic linkages between epipelagic and mesopelagic depths) (Frid et al. 2009). In the case of exploited marine species, knowledge about vertical movement behavior is also essential to understand their interaction with commercially important fishes and with fisheries gear which is deployed at different depths depending on target species and gear type (e.g., Goodyear et al. 2008; Beverly et al. 2009). With sharks in particular, reducing susceptibility to fisheries targeting other species is a major goal given the large number of sharks caught as bycatch (reviewed by Barker and Schluessel 2005), and information on their vertical behavior may help this effort (Beverly et al. 2009; Musyl et al. 2011).

Tiger sharks (*Galeocerdo cuvier*) are large sharks found in tropical and subtropical waters around the world and are associated with coastal and pelagic habitats. In these systems, tiger sharks may play an important ecological role by influencing the behavior of their prey, which may result in behaviorally mediated trophic cascades that can ultimately affect primary producers (Heithaus et al. 2012; Burkholder et al. 2013). Given the potential impact of tiger sharks in marine systems, knowledge of their movements is important for species management as well as understanding ecosystem function, especially considering tiger sharks appear to have experienced population declines in some areas (Baum et al. 2003; Myers et al. 2007; Holmes et al. 2012).

Indeed, tiger shark movements have been the focus of intensive study in Hawaii and Australia (e.g., Holland et al. 2001; Heithaus et al. 2002; Meyer et al. 2009, 2010; Papastamatiou et al. 2011; Fitzpatrick et al. 2012; Papastamatiou et al. 2013; Werry et al. 2014), and their horizontal movement patterns appear to be highly variable at local and large scales. Acoustic telemetry has revealed movements between islands within the Hawaiian Archipelago (Holland et al. 1999; Meyer et al. 2010; Papastamatiou et al. 2013), while tag and recapture data and satellite telemetry have revealed long distance and even transoceanic movements (Kohler and Turner 2001; Heithaus et al. 2007; Meyer et al. 2010; Shivji and Wetherbee unpublished data). Some movements even appear to be influenced by seasonal pulses in prey availability (Simpfendorfer et al. 2001; Wirsing et al. 2007; Meyer et al. 2010).

There is far less information available on tiger shark vertical movements. Tiger sharks in Hawaii tracked by acoustic telemetry in waters <150 m deep tended to be associated with the substrate (Holland et al. 1999; Nakamura et al. 2011). When encountering deeper waters, these sharks adopted yo-yo dives (repeated oscillatory dives; Klimley et al. 2002) in the upper 100 m of the water column and occasionally made deep dives (>200 m). These observations, however, were short in duration (<50 h). Satellite telemetry-based tracks of tiger sharks in Hawaii and Australia lasting days to several months also revealed deep diving behavior (500–1100 m) (Meyer et al. 2010; Fitzpatrick et al. 2012; Werry et al. 2014), but the resolution of the depth data did not allow for a fine-scale investigation of the vertical dive patterns.

Here, we describe the vertical habitat use of tiger sharks from two regions of the western North Atlantic Ocean (the northern Caribbean Sea, specifically the area around the Puerto Rico-Virgin Islands platform, and Bermuda), inferred from pop-up satellite archival tags. Our goals were to (1) quantify tiger shark depth and temperature distributions and dive patterns, (2) investigate whether tiger sharks display consistent patterns of vertical habitat use, and (3) investigate potential factors that might influence vertical behavior.

## Materials and Methods

### Shark tagging

Tiger sharks were captured within the U.S. Virgin Islands (USVI) in March and June 2008 and at Bermuda in August and October 2009. In the USVI, sharks were caught using bottom longlines set at depths of 20–40 m and allowed to soak for 3–4 h. Longlines were 366 m long with 25 360-cm gangions terminating in a 16/0 recurved hook. Sharks were caught using rod and reel in Bermuda. At both locations, fishing occurred on mesophotic reefs close to the edge of the insular platform. Captured sharks were measured for fork length, sexed, fitted with satellite transmitters, and released.

Fourteen (USVI: nine; Bermuda: five) tiger sharks were tagged with pop-up satellite archival tags [either a PTT-100 High Rate (HR) tag or PTT-100 standard tag: 166 × 41 mm; Microwave Telemetry Inc, Columbia, MD]. Tags were attached to an umbrella dart via 20 cm of 900-lb test monofilament leader encased in surgical tubing. The tags were affixed to the sharks by anchoring the dart into the dorsal musculature lateral to the first dorsal fin (Domeier et al. 2005).

All tags recorded and archived depth (HR tag: ±1.3 m; and standard tag: ±5.4 m), temperature (±0.23°C), and light levels (<4 × 10^−5^ Lux @ 555 nm) at set intervals and were programed to detach from the shark after periods ranging from 23 days to 12 months. Once detached, the tags float to the surface and transmit the archived raw data (i.e. the individual data points) via satellite uplink. Because of limitations in battery life and satellite coverage, typically only a subset of the archived data is successfully transmitted. Because of the higher data recording rate of HR tags (a consistent interval between 3.5 and 4 min depending on the individual tag), HR tag deployments were limited to 23–28 day. Standard tags, which were programmed for 6–12-month deployments, initially record data every 15 min. After 4 months, standard tags begin recording data at 30-min intervals, overwriting data stored at 15-min intervals, and after 8 months, data are recorded hourly overwriting data stored at 30-min intervals.

### Data analysis

Transmitted depth and temperature time-series data were split into periods of daytime and nighttime based on times of sunrise and sunset (http://aa.usno.navy.mil/data). For sharks tagged with the standard tags, sunrise and sunset were determined from the estimates of daily locations of the most probable track calculated from tag-recorded light data using a Kalman filter state-space model (kftrack package) in R (R Development Core Team; The R Project) (Sibert et al. 2003), as opposed to estimates of sunrise and sunset times provided from the tags. For sharks tagged with HR tags, times of sunrise and sunset were interpolated on the basis of the times of sunrise and sunset at the tagging and tag pop-up locations. Positional estimates were not calculated for HR tags, which store light data at a lower resolution than standard tags in order to allow archiving of depth and temperature at more frequent intervals to obtain higher resolution information on these parameters.

All sharks appeared to display irregular dive behavior (i.e., early postrelease dive behaviors and depth use inconsistent with the remainder of the track) for a few days immediately after tagging. These periods (2–7 days) of irregular behavior were not used for analysis. We created histograms of individual depth and temperature readings to examine the vertical and thermal distributions of each shark. Because the animals were tagged in different seasons and locations, we also created histograms of the temperatures experienced by sharks standardized in reference to estimated daily sea surface temperature (SST) derived from the tag. For these standardizations, we used the highest temperature recorded by the tag on a given day (sunrise to the following sunrise) as a proxy for sea surface temperature. In all instances, the highest daily temperature was observed when the tags were recording a depth of 0.0 m. For each shark, we compared depth and temperature distributions from daytime and nighttime periods using a two-sample Kolmogorov–Smirnov test. Prior to the Kolmogorov–Smirnov tests, each shark's daytime and nighttime periods were tested for autocorrelation. Data from sharks were subsampled at the first lag that resulted in a correlation coefficient <0.2, or ≤0.3 for the two sampling periods lacking a lag with a correlation coefficient <0.2.

To examine dive periodicity in tiger sharks, we applied a fast Fourier transformation (FFT) (Chatfield 2004) with Hamming window smoothing in SigmaPlot 11.0 (Systat Software, Inc., San Jose, CA) to the time series of depth measurements of each shark. Smoothing using a Hamming window reduces the effects of adjacent spectral components, which can generate biologically meaningless frequency peaks. The FFT requires data sampled at a regular time interval; however, our datasets were scattered with gaps of various lengths resulting from partially transmitted tag data received via satellite (Table 1). These gaps were filled using a shark's mean depth calculated from the shark's entire useable transmitted dataset (i.e., periods of initial irregular dive behavior not included) under the rationale that the filled gaps in the dataset should represent only noise in the dataset (i.e., no periodic signal), making it harder to detect dive periodicities, and is therefore a conservative approach. Prior to performing the FFT on the actual depth data, we performed an FFT on the presence and absence of data to examine whether there were periodicities to the gaps in the dataset. No periodicities in the dataset gaps were detected ensuring that by filling in the gaps, we did not introduce any artificial periodic components that would be detected by the FFT.

**Table 1:** Summary information for tiger sharks, *Galeocerdo cuvier*, tagged with Pop-up Satellite Archival Tags in the US Virgin Islands and Bermuda

Shark number	Sex	Fork length (cm)	Maturity	Tag type	Date tagged	Days at liberty	Net displacement (km)	% data received
USVI								
1	F	290	Likely mature	HR tag	21 March 2008	28	1147	66
2	F	266	Likely mature	Standard tag	22 March 2008	33	396	58
3	F	233	Immature	Standard tag	23 March 2008	158	347	100^1^
4	M	287	Likely mature	HR Tag	3 June 2008	26	100	72
5	M	224	Immature	HR Tag	4 June 2008	9	103	83
6	M	207	Immature	HR Tag	4 June 2008	–	–	–2
7	M	290	Likely mature	HR Tag	5 June 2008	26	24	34
8	F	210	Immature	HR Tag	6 June 2008	16	83	19
9	F	244	Immature	HR Tag	6 June 2008	–	–	–2
Bermuda								
10	M	277	Likely mature	Standard tag	2 August 2009	–	–	–2
11	M	259	Likely mature	Standard tag	3 August 2009	–	–	–3
12	M	262	Likely mature	Standard tag	3 August 2009	183	1354	2^4^
13	M	305	Likely mature	Standard tag	5 August 2009	184	1164	92
14	M	277	Likely mature	Standard tag	3 October 2009	47	1181	88

1 Tag recovered.

2 Tag did not report.

3 Tag possibly eaten shortly after deployment.

4 Tag not analyzed because of insufficient data return.

Because of variability in the depth distributions among the tiger sharks, we grouped individuals into behavioral types using cluster analysis (Jorgensen et al. 2012). Depth bins with values <0.05 were set to 0 to de-emphasize rare events, and Manhattan distances between each shark were calculated. Single-linkage hierarchical cluster analysis was performed on the distance matrix using PRIMER 6 (PRIMER-E Ltd, Lutton, UK).

## Results

### Tag results

Ten of the 14 deployed tags transmitted data via satellite resulting in the retrieval of 2–92% of their archived data; additionally, one tag was recovered allowing retrieval of 100% of the its archived data (Table 1). One of the 10 transmitting tags did not provide enough usable data for analyses and, inferring from the light data obtained, another transmitting tag appeared to have been eaten shortly after deployment. This resulted in usable data from nine sharks in total (USVI: seven; Bermuda: two). For the seven reporting sharks tagged in the USVI, track durations for five sharks tagged with HR tags ranged from 9 to 28 day. Overall, 1161 to 6957 depth and temperature readings were received from these tracks. Track durations for two USVI sharks tagged with standard tags were 33 and 158 days (1020 depth/1242 temperature readings and 9737 depth/9959 temperature readings, respectively). The seven USVI reporting tags collectively resulted in 298 days of data (30,506 depth and 30,728 temperature readings). Two Bermuda-tagged sharks providing usable depth and temperature data had track durations of 47 and 184 days (3943 depth/3930 temperature and 11,276 depth/11,007 temperature readings, respectively), giving a total of 231 days of data (15,219 depth and 14,937 temperature readings; Table 1).

### USVI shark movements

**Figure 1:**
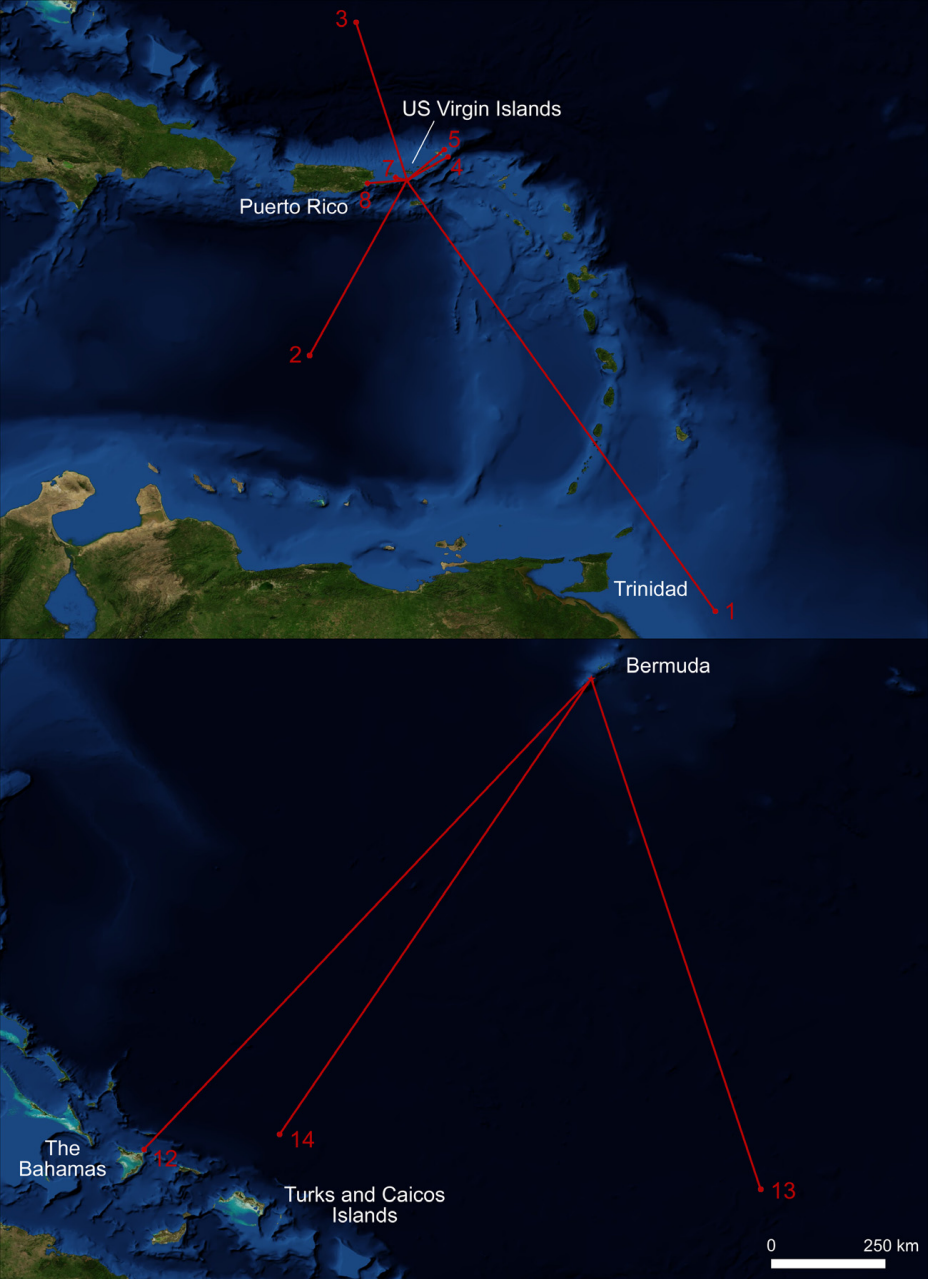
Net displacement for tiger sharks tagged in the US Virgin Islands (upper panel) and Bermuda (lower panel). Numbers correspond to shark number from Table 1.

Tiger sharks tagged in the USVI ranged in size from 207 to 290 cm FL and had net horizontal displacements (i.e., the distance between tagging and pop-up locations) of 24–1147 km (Table 1; Fig. 1). Tag pop-up locations for three of the seven sharks (sharks #1, #2, and #3) indicated that these sharks moved off the Puerto Rico–Virgin Islands platform during the course of their tracks (28–158 days at liberty), with shark #1 ultimately being detected ∼240 km east of Trinidad and Tobago. Four sharks (sharks #4, #5, #7 and #8) had their HR tags pop-up over (sharks #7 and #8; 28 and 16 days at liberty, respectively) or within 10 km of the platform (sharks #4 and #5; 26 and 9 days at liberty, respectively). Depth data from these sharks indicate that they also spent time off the platform, with sharks #4, #5, and #7 regularly spending time off the platform.

**Figure 2:**
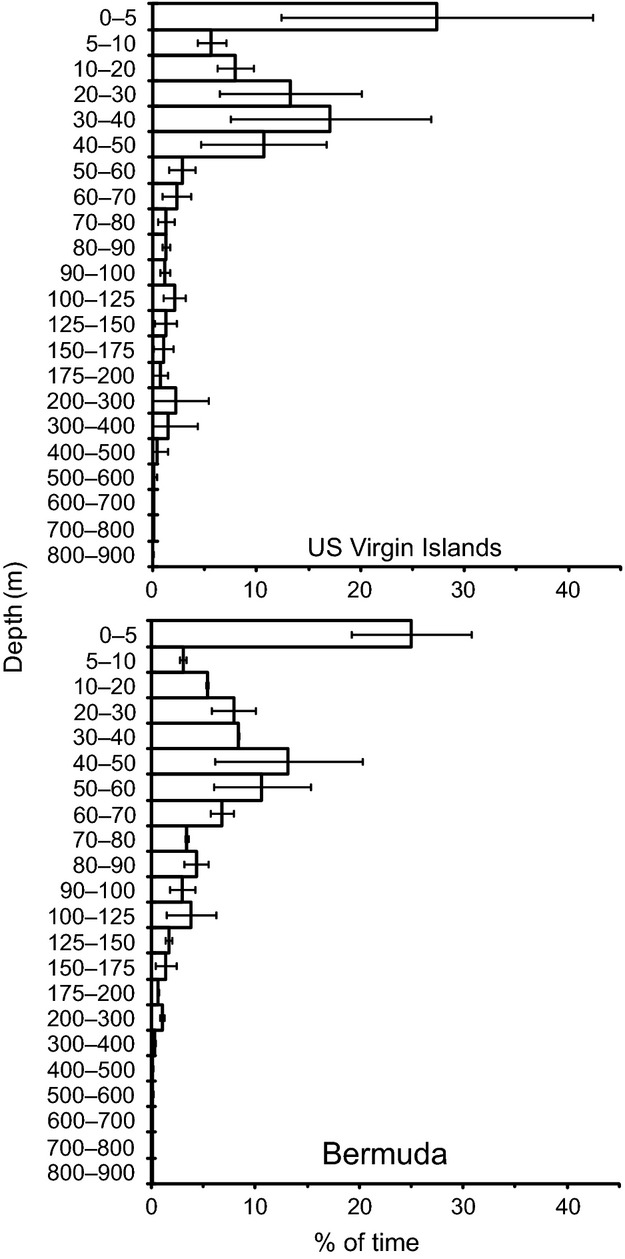
Overall depth distribution (mean ± SD) of seven tiger sharks tagged in the US Virgin Islands and two tiger sharks tagged in Bermuda.

All seven USVI-tagged sharks displayed yo-yo dives for the duration of their tracks, and made multiple dives to depths greater than 200 m with maximum depths ranging from 263.6 m to 718.2 m (Table 2). Sharks spent 81.9 ± 12.8% (mean ± SD; range: 56.0–93.2%) of their time in the upper 50 m of the water column (Fig. 2). Sharks experienced temperature ranges as low as 6.3°C (from 20.4 to 26.7°C) and as high as 20.3°C (from 8.9 to 29.1°C; Table 2). Overall, USVI-tagged sharks used a relatively small range of temperatures, with an average of 81.1 ± 14.1% (range: 55.1–99.5%) of their time spent in waters within 2°C of SST.

**Table 2:** Depth and temperature parameters for nine tiger sharks tagged in the US Virgin Islands (USVI) and Bermuda (BMD), categorized by behavioral type (see Results section for description of behavioral types). Values are median (1st quartile–3rd quartile)/maximum depth or minimum temperature. An * after daytime medians indicates a significant difference between daytime and nighttime distributions subsampled to account for autocorrelation (two-sample Kolmogorov–Smirnov test, *P* < 0.05)

Shark number	Dates tracked	Depth (m)		Temperature (°C)	
	
		Daytime	Nighttime	Daytime	Nighttime
Surface-oriented					
1 (USVI)	21 March to 16 April 2008	2.7* (0.0–21.5)/418.3	26.9 (0.0–57.8)/442.5	26.0* (25.1–26.6)/12.4	25.8 (23.1–26.4)/10.7
5 (USVI)	4 June to 13 June 2008	1.3* (1.3–9.4)/166.8	41.7 (1.3–100.9)/450.5	28.2* (27.8–28.4)/21.5	26.9 (27.8–28.4)/16.2
Bimodal-shallow					
2 (USVI)	22 March to 23 April 2008	26.9 (10.8–32.3)/166.8	26.9 (5.4–43.0)/263.6	26.0 (25.8–26.2)/20.5	26.0 (25.8–26.2)/20.4
3 (USVI)	23 March to 28 August 2008	32.3* (10.8–43.0)/317.4	32.3 (21.5–37.7)/392.7	27.1 (26.2–28.2)/17.2	27.1 (26.2–28.2)/16.0
7 (USVI)	5 June to 2 July 2008	37.7* (16.1–45.7)/406.2	25.6 (1.3–39.0)/426.3	27.3* (26.4–28.0)/16.2	28.2 (27.3–28.4)/16.0
8 (USVI)	6 June to 22 June 2008	29.6* (18.8–36.3)/406.2	22.9 (2.0–37.7)/555.4	28.0 (27.3–28.4)/14.9	28.2 (27.3–28.6)/12.6
14 (BMD)	3 October to 19 November 2009	38.3* (8.1–53.1)/601.2	43.0 (17.5–59.8)/291.8	26.4* (25.8–26.9)/14.8	26.0 (25.0–26.7)/14.8
Other					
4 (USVI)	3 June to 29 June 2008	63.2* (20.2–234.0)/613.3	33.6 (5.4–67.2)/718.2	26.2* (19.7–27.8)/11.0	27.5 (26.2–28.2)/8.9
13 (BMD)	5 August 2009 to 5 February 2010	32.3* (0.0–69.9)/828.4	43.0 (5.4–75.3)/726.2	24.8* (23.7–26.2)/12.0	24.8 (23.7–26.0)/10.3

Although all USVI-tagged sharks spent the majority of their time shallower than 50 m and typically experienced a narrow range of temperatures, we note that diving behavior and depth distributions differed greatly among individuals. Additionally, there was no clear association of these interindividual differences to sex, size, season, or horizontal displacement of the animals (see below).

**Figure 3:**
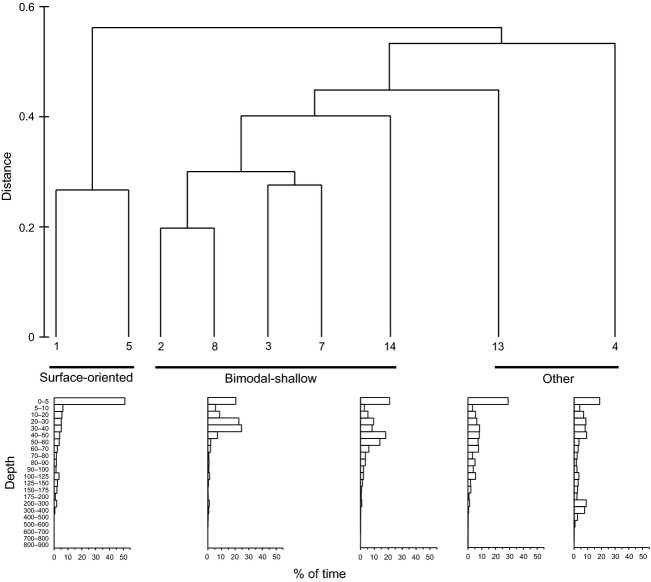
Single-linkage cluster analysis of the depth distributions of tiger sharks identifying behavioral types. Histograms show representative examples from each behavioral type.

**Figure 4:**
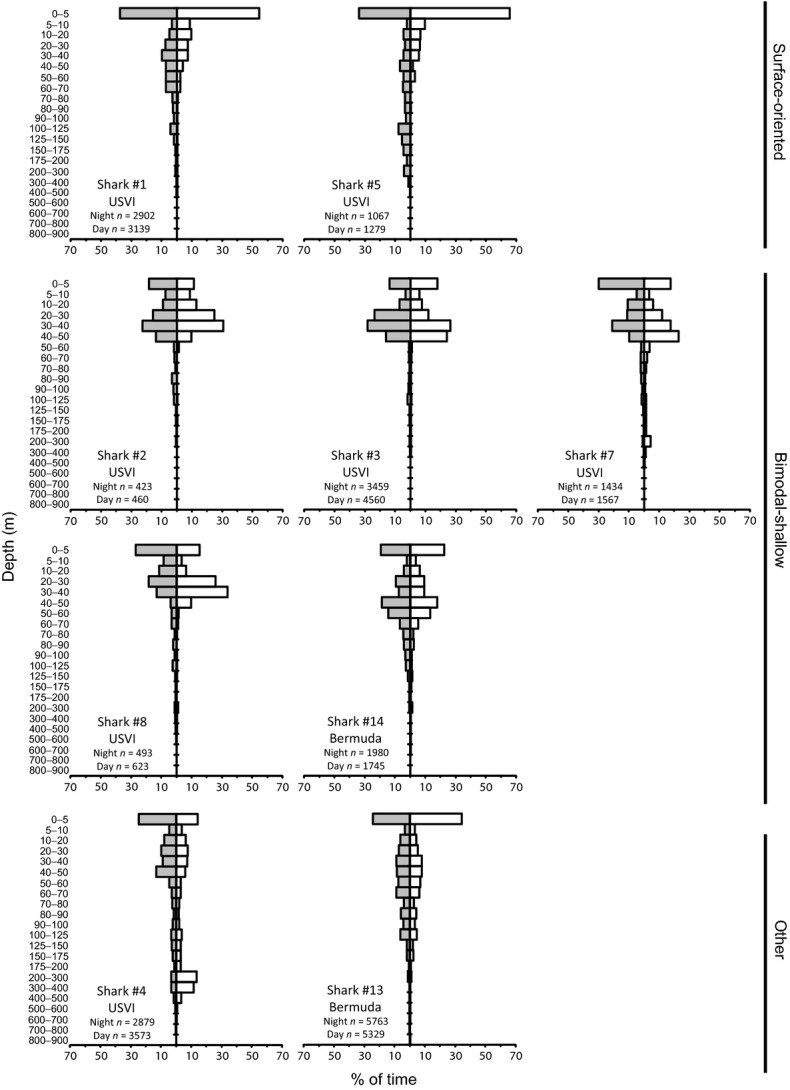
Daytime (white) and nighttime (gray) depth distributions of tiger sharks by behavioral type. *n* is the total number of depth reading. Daytime and nighttime depth distributions subsampled to account for autocorrelation differed significantly for all sharks (two-sample Kolmogorov–Smirnov test, *P* < 0.05), except shark #2.

Despite the interindividual variability in vertical habitat use, cluster analysis identified two behavioral types in the USVI-tagged sharks on the basis of depth distribution (Fig. 3). The first behavioral type was observed in two sharks (sharks #1 and #5) that spent large amounts of time near the surface (hereafter “surface-oriented” sharks). These sharks spent approximately half (46–51%) their time at <5 m (Fig. 4). In contrast, four sharks (sharks #2, #3, #7, and #8) had primarily bimodal depth distributions, spending 15–23% of their time at <5 m and 47–65% of their time at 20–50 m (Fig. 4), and only 2–8% time below 100 m depth (hereafter “bimodal-shallow” sharks). The remaining shark (shark #4) did not fit either behavioral type, spending 19% of its time at <5 m, 26% of its time between 20 and 50 m, but a substantial portion of its time (31%) at >100 m, including 19% of its time between 200 and 500 m (Fig. 4).

**Figure 5:**
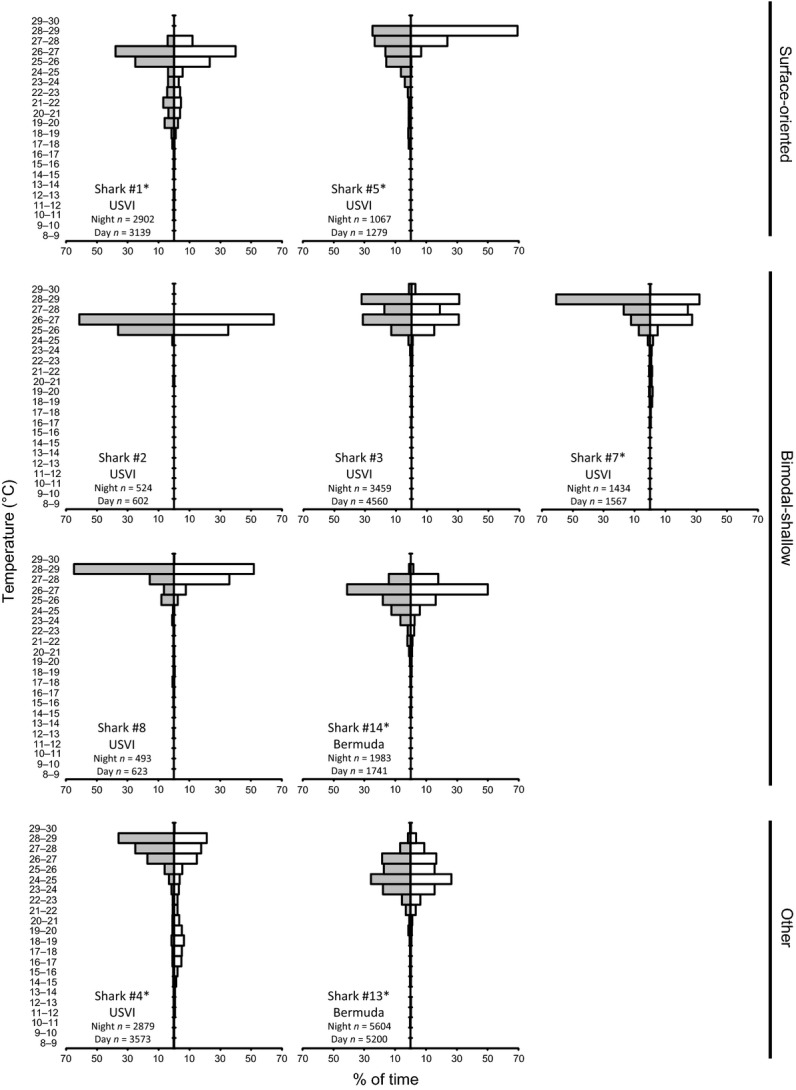
Daytime (white) and nighttime (gray) temperature distributions of tiger sharks by behavioral type. *n* is the number of temperature records. Asterisks indicate differences between daytime and nighttime distributions subsampled to account for autocorrelation (two-sample Kolmogorov–Smirnov test, *P* < 0.05).

**Figure 6:**
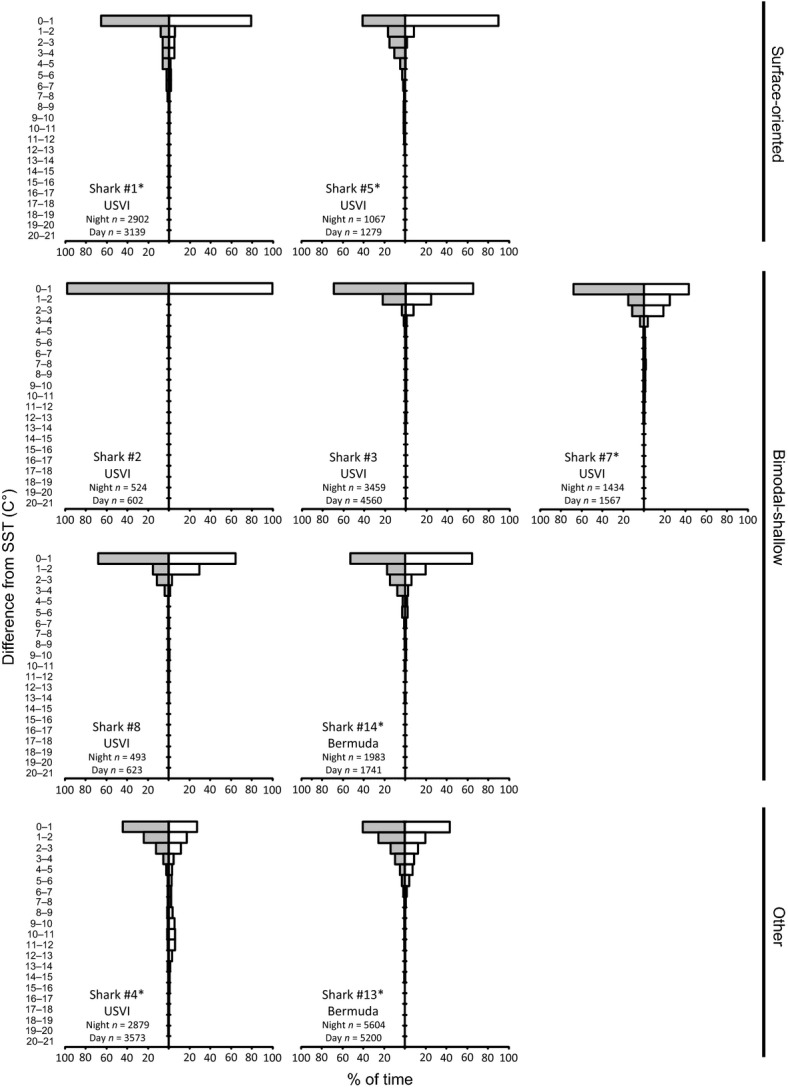
Daytime (white) and nighttime (gray) distributions of temperatures scaled to sea surface temperature (SST) of tiger sharks by behavioral type. *n* is the number of temperature records. Asterisks indicate differences between daytime and nighttime distributions subsampled to account for autocorrelation (two-sample Kolmogorov–Smirnov test, *P* < 0.005).

**Figure 7:**
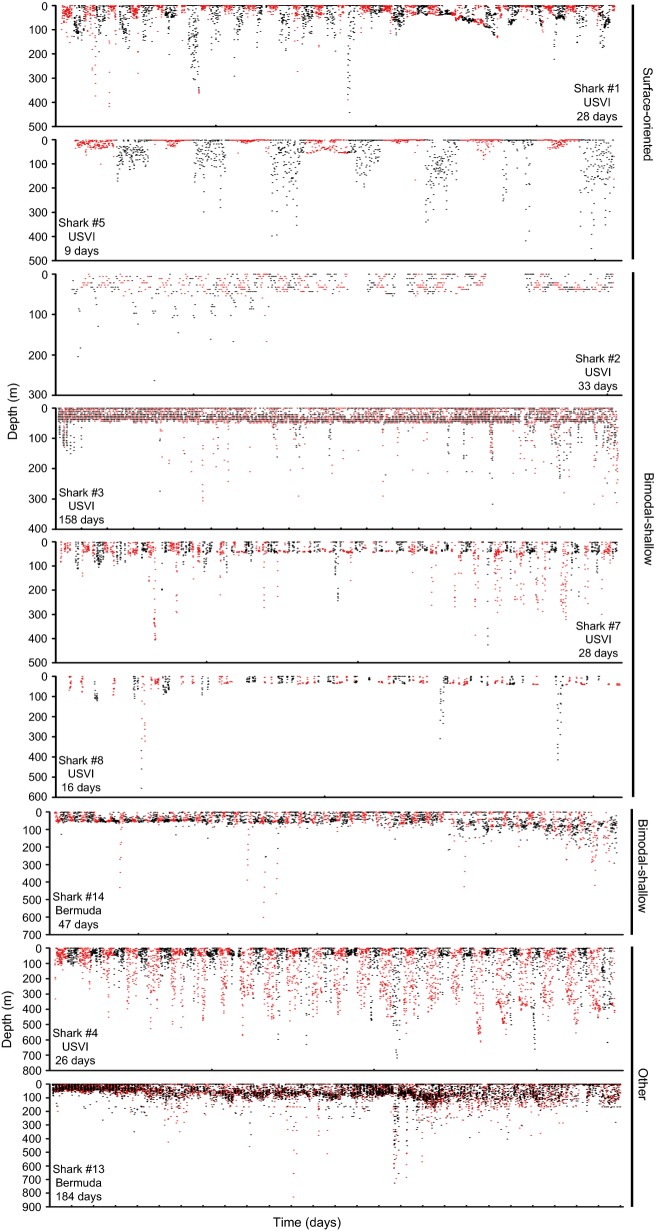
Dive profiles (daytime: red and nighttime: black) of tiger sharks by behavioral type. Tick marks along the time axis are at 7-day intervals.

Both surface-oriented sharks displayed similarities in their vertical behavior, despite striking differences in their horizontal movements. Shark #1 (a 290 cm FL female tagged in March) travelled over 1100 km over the course of 28 days, leaving the Caribbean Sea, whereas, shark #5 (a 224 cm FL male tagged in June) had a net displacement of ∼100 km in 9 days (Fig. 1). Both these sharks had a 24-h periodicity in their depths (determined by FFT analysis) and depth distributions that differed between daytime and nighttime (*P* < 0.001, Fig. 1), with more time spent at depth during the nighttime. Similarly, distributions of water temperature and temperature standardized to SST differed between daytime and nighttime (all *P* < 0.05; Figs. 5, 6), with warmer temperatures experienced during the daytime. Both sharks exhibited similar fine-scale dive profiles during their tracks, making frequent deep dives throughout the nighttime, while rarely diving deeper than 50 m during the daytime (Fig. 7).

There were overall similarities but also individual variability in the vertical behavior of the four bimodal-shallow sharks. Three of the four sharks had spectral peaks corresponding to a 24-h period (all but shark #8), although the magnitude of the peaks were low for sharks #2 and #3 indicating the behavior giving rise to the peaks was infrequent. Daytime and nighttime depth distributions differed for three of the bimodal-shallow sharks (sharks #3, #7, #8; all *P* < 0.05; Fig. 4), further supporting diel periodicity. Sharks #7 and #8 spent more time near the surface during the nighttime. In sharks #2 and #3, the deeper modal peaks differed between daytime and nighttime. For shark #2, the peak was slightly shallower during the daytime, while in shark #3, the peak was broader and centered at a shallower depth during the nighttime. Daytime and nighttime distributions for water temperature and temperature standardized to SST differed significantly for shark #7 (*P* < 0.005; Figs. 6, 7). This shark tended to experience warmer temperatures during the nighttime.

Although overall depth distributions of the bimodal-shallow sharks were similar, examination of their fine-scale dive profiles (Fig. 7) revealed that this group lacked behavioral uniformity in this context, with individuals differing markedly in their dive behaviors. Bimodal-shallow sharks tended to make less frequent deep dives than surface-oriented sharks, but the timing and frequency of diving differed between sharks. Sharks #2 and #8 tended to make deep dives during the nighttime, while sharks #3 and #7 tended to make deep dives during the daytime (Fig. 7). Both groups (daytime and nighttime divers) included sharks that left (sharks #2 and #3) and remained in the general vicinity of the Puerto Rico–Virgin Islands platform, or at least returned to it (sharks #7 and #8).

The single shark (shark #4, a 287 cm FL male; 27-day track) whose depth distribution did not fall into either category (Fig. 3) also displayed a clear diel periodicity with a large spectral peak corresponding to a 24-h period. Consistent with a diel periodicity, depth distribution differed between daytime and nighttime (*P* < 0.001; Fig. 4) with more time at depth during the daytime compared with the nighttime. Distributions of water temperature and temperature standardized to SST differed between daytime and nighttime (both *P* < 0.001; Fig. 6), with warmer temperatures experienced during the nighttime. This shark made frequent deep dives throughout daytime and nighttime periods over the course of its whole track. Although its deepest dives occurred at nighttime, deep dives tended to be deeper and more frequent during daytime periods, with 78.3% of depth readings >300 m occurring during the daytime (Fig. 7).

### Bermuda shark movements

Tiger sharks tagged in Bermuda ranged in size from 259 to 305 cm FL, and in contrast to most USVI sharks undertook large-scale movements showing net horizontal displacements of 1164–1354 km (Table 1; Fig. 1). The two sharks (sharks #13 and #14) that provided usable data left Bermuda shortly after tagging and remained in pelagic waters up to 6000 m deep for the durations of their tracks.

Like the USVI-tagged sharks, the two Bermuda-tagged sharks (shark #13–184-day track; shark #14–47-day track) made yo-yo dives throughout their tracks; they made multiple dives to depths greater than 200 m (Fig. 7) with maximum recorded depths of 828.4 m and 601.2 m, both of which occurred in the daytime (Table 2). These sharks also spent the majority of their time at depths <50 m (60.76% and 64.91% of their time, respectively; Fig 2). These sharks experienced temperature ranges of 19.4°C (from 10.3 to 29.7°C) and 13.8°C (from 14.8 to 28.6°C; Table 2). Most of their time, however, was spent within 2°C of SST (64.6%, and 76.9%, respectively).

Although showing some mild similarities to the surface-oriented sharks, shark #13 did not cluster within this behavioral type (Fig. 3). Like surface-oriented sharks, shark #13 displayed a unimodal depth distribution with the highest frequency at <5 m, but only 29% of its time was spent at these shallow depths (Fig. 4) in contrast to the surface-oriented sharks (46–51% of time). The depth use distributions between 5 m and 100 m for shark #13 were fairly uniformly distributed, accounting for 59% of the shark's overall tracked time. In contrast to all but one of the USVI sharks, FFT analysis did not reveal 24-h periodicity, although depth distributions differed between daytime and nighttime (*P* < 0.001; Fig. 4), with more time spent at the surface during the daytime than at nighttime. Further, distributions of water temperature and temperature standardized to SST differed between daytime and nighttime (all *P* < 0.025; Fig. 6), with warmer temperatures experienced during the daytime. Overall, shark #13 was not limited to the upper 50 m of the water column to the degree observed in USVI-tagged sharks, and despite making deep dives throughout its track, did not display a clear pattern of deep diving periodicity, with deep dives occurring both during the daytime and nighttime (Fig. 7).

Despite some differences in its depth distribution, shark #14 was classified as a bimodal-shallow shark because it clustered most closely with and showed overall similarities to the depth distributions of the other sharks in this category (Fig. 3). Shark #14 spent a similar amount of time at <5 m (21%) as USVI bimodal-shallow sharks (15–23%), and displayed a second deeper peak, like USVI bimodal-sharks; however, shark #14's deeper peak in the depth distribution was centered at a slightly deeper depth around 40–60 m, as opposed to 20–50 m as observed in bimodal-shallow sharks from the USVI (Fig. 4). For shark #14, FFT analysis also failed to detect 24-h periodicity, although depth distributions differed between daytime and nighttime (*P* < 0.001; Fig. 4), with slightly more time spent at shallower depths during the daytime. Consistent with differences in depth distributions, water temperature and temperature standardized to SST distributions differed between daytime and nighttime (all *P* < 0.001; Fig. 6), experiencing warmer temperatures during the daytime. Shark #14 was an infrequent deep diver with deeper dives primarily taking place during the daytime (Fig. 7).

## Discussion

Tiger sharks tracked in the Caribbean and western North Atlantic showed a number of broad similarities in overall vertical habitat use, but also striking differences in the vertical movements among individuals apparently unrelated to sex, size, horizontal movements, or the physical environment. All tiger sharks tracked demonstrated a high frequency of yo-yo diving within the isothermal layer, with sharks typically remaining in the upper 50 m of the water column, but making multiple dives to depths >200 m. Most USVI- and Bermuda-tagged sharks also spent a notably large amount of time at shallow depths (upper 5 m). The observations of yo-yo diving behavior primarily in the upper water column, interspersed with deeper dives have also been observed in the few other studies that have examined tiger shark vertical movements in other parts of the world. Such shared vertical movement features in sharks tagged at different locations and exhibiting widely varying horizontal displacements suggests that these vertical behaviors are a common behavioral trait in tiger sharks. This idea is supported by examination of tiger shark depth distributions and vertical behaviors in the Hawaiian Islands and northern Australia. Acoustic and satellite telemetry and accelerometers have revealed similar dive patterns to those we observed from the USVI and Bermuda. Hawaii-tagged tiger sharks conducted yo-yo dives in the upper 100 m of the water column and occasional dives to >200 m were also observed (Holland et al. 1999; Nakamura et al. 2011). Pop-up satellite archival telemetry conducted in the Northwestern Hawaiian Islands and Australia, although lacking the temporal resolution to elucidate yo-yo dives, also showed that sharks typically used the upper 100 m of the water column and that dives to >200 m were common with sharks from both locations (Meyer et al. 2010; Fitzpatrick et al. 2012; Werry et al. 2014). However, in contrast to the sharks tagged along the insular platforms in the USVI and Bermuda, sharks tagged off Hawaii and Australia spent far less time in the upper 5 m of the water column (Holland et al. 1999; Fitzpatrick et al. 2012).

Although at a coarse-scale, sharks tagged in the USVI and Bermuda displayed general similarities in their vertical behaviors (i.e., yo-yo diving mainly within the upper 50 m and a substantial portion of time spent in the upper 5 m), examination of detailed vertical movements showed that individuals engaged in distinctly different behaviors. With some success, sharks could be categorized into general groups on the basis of depth distribution, although there was variability even within these groups. Surface-oriented sharks spent approximately half their time in the upper 5 m of the water column. Sharks in the bimodal-shallow category were in the upper 5 m for ∼20% of the time with an additional peak in activity between 20 m and 60 m. Two sharks did not fit these categories; one shark spent ∼20% of its time within the top 5 m of the water column and ∼20% of time between 200 m and 500 m, while the other shark spent ∼30% of its time at depths of <5 m and displayed fairly even use of waters between 5 m and 100 m.

Such differences in behavior can often be related to characteristics of the animals, such as sex, age, or migratory behavior (e.g., Lukoschek and McCormick 2001; Boustany et al. 2002; Beck et al. 2003), but such characteristics do not appear to be driving behavioral differences in the tiger sharks in this study. Acknowledging the limitation that the sample size of each sex in our study is small (four female and five male sharks), the sharks did not segregate into depth distributions by sex, with male sharks belonging to both depth distribution categories and making up both individuals that did not fit into categories. Females also belonged to both depth distribution categories. Similarly, on the basis of size, both mature (FL > ∼260 cm) and immature animals occurred in both surface-oriented and bimodal-shallow depth categories. Further, horizontal movement undertaken by the sharks (short vs. long horizontal displacements) also did not correspond with depth distribution. Displacements of <100 km and >350 km were each observed for individuals from both depth distribution categories.

Examination of both behavioral types at finer scales revealed that sharks belonging to a behavioral type did not necessarily engage in similar dive behaviors. Typical dive depths differed among group members for both of these depth distribution categories. Bimodal-shallow sharks, however, showed far greater variety in their dive behaviors than surface-oriented sharks. Bimodal-shallow sharks varied greatly in the frequency of deep diving behavior over short and long time periods. Daily timing of deep dives also varied among bimodal-shallow sharks, with three sharks primarily performing deep dives during the daytime and two sharks performing these dives mainly during the nighttime. The variability observed in bimodal-shallow sharks suggests a cautionary perspective when interpreting vertical habitat use solely on the basis of depth distributions, because animals with similar depth distributions could be using the water column quite differently; therefore, depth distributions alone may inadequately elucidate vertical habitat use patterns.

Behavioral variability has often been attributed to differing environmental conditions (e.g., Sims et al. 2005; Queiroz et al. 2012). Examination of several of these factors, however, failed to explain vertical habitat use and the variability observed among tiger sharks. At the broad scale, for example, there was no obvious connection between behavioral type and season, with surface-oriented and bimodal-shallow sharks tagged across multiple months (March and June; and March, June and October, respectively) or between behavioral type and tagging location, with bimodal-shallow sharks tagged in the USVI and Bermuda. At finer scales, thermal gradients are one factor that can influence vertical movement patterns. For example, many fishes in the pelagic environment limit the majority of their movements to the isothermal layer (e.g., Walli et al. 2009; Weng et al. 2009; Chiang et al. 2011). Tiger sharks, however, typically experienced a water column that was isothermal to depths of 80–100 m and most sharks spent a very large proportion of their time at depths well above the lower boundary of the isothermal layer. In addition, the lower boundary of the isothermal layer was deeper than the self-imposed depth floor displayed by tiger sharks when not making deep dives, so thermal gradients do not explain differing vertical habitat use. Although temperature does not appear to be a major factor limiting the depth of yo-yo dives in the upper water column, it may influence the time tiger sharks spend at depth. During deep dives tiger sharks often experienced temperature changes of >8°C and the deep dives tended to be short in duration. Oxygen levels, which are also known to limit dive behavior in pelagic species (Prince and Goodyear 2006; Prince et al. 2010), likely did not provide a barrier to the depth of tiger shark in this study because oxygen levels throughout the water column in these parts of the western North Atlantic Ocean are higher than levels that are thought to restrict the diving behavior of shortfin mako and white sharks movements (Nasby-Lucas et al. 2009; Garcia et al. 2010; Abascal et al. 2011), which have higher metabolic rates than ectothermic sharks (Sepulveda et al. 2007; Bernal et al. 2012) and therefore higher oxygen requirements.

Dive behaviors could also be a means of orientation. It has been suggested that sharks, including tiger sharks, use cognitive maps of their home ranges to orient at various spatial scales (Meyer et al. 2010; Papastamatiou et al. 2011). Tiger sharks may use deep dives to find their bearings. In fact, deep dives by tiger sharks in Hawaii were infrequent and tended to occur as sharks were leaving or approaching shallow banks (Holland et al. 1999). Sharks #7 and #8 in our study showed limited horizontal displacement over their tracks, and immediately before or after deep dives were often associated with depths consistent with the edge of the platform. It is therefore feasible that deep dives by these individuals could coincide with movements off of or on to the platform, as observed in Hawaii. Another hypothesis suggests deep dives may be useful in navigation on the basis of differences in the Earth's magnetic field across depths (Klimley et al. 2002). We observed deep dives of varying frequency by tiger sharks engaged in long-distance directional movements as well as relatively local movements. The high frequency of deep dives by some sharks (e.g., sharks #4 and #5) that appeared to stay in the vicinity of the Puerto Rico–Virgin Islands platform suggests that navigation via magnetic fields was not the primary reason for these dives. Although this does not preclude navigation via magnetic fields as a factor in the deep diving behavior of tiger sharks making long-distance directional movements, such navigational deep dives have been associated with sunrise and sunset (Willis et al. 2009), which was not observed in any of the tiger sharks.

Although physical factors may have some influence on the vertical distribution of tiger sharks, and yo-yo diving may be an energetically efficient means of maximizing horizontal distance travelled (Iosilevskii et al. 2012), foraging behavior may also be an explanation for many of the observed vertical movements and interindividual variability. Tiger sharks have a broad dietary breadth, feeding on fish, invertebrates, marine mammals, marine reptiles, and birds (Lowe et al. 1996; Simpfendorfer et al. 2001), suggesting plasticity in foraging behaviors. Given this potential plasticity and the low productivity of environments such as tropical waters and the open ocean, it is possible that in response to low prey abundance tiger sharks may diversify their dive behaviors as individuals target different prey resources (Tinker et al. 2007, 2008). Furthermore, even if individual sharks are not targeting different prey, variability in dive behaviors could arise in response to patchy prey distributions, which are common in pelagic waters. Searching the water column for patchy prey could explain the variability and lack of a pattern seen in the deep diving behavior among bimodal-shallow sharks. We recognize, however, that depth limitations when sharks were over the Puerto Rico–Virgin Islands platform (30–50 m depth) could also have obscured deep diving behavioral patterns.

Periods of deeper dives may represent an expanding of the foraging arena when shallow water prey is scarce. The presence of deep water crabs in the stomachs of tiger sharks (Rancurel and Intes 1982; J. J. Vaudo, pers. obs.) confirms that tiger sharks do feed in deeper waters, and such prey items are likely found on the slope of the Puerto Rico–Virgin Islands platform. A shift from extended periods above the thermocline to periods of frequent dives below the thermocline in common thresher sharks has been interpreted as a response to regional differences in prey availability (Cartamil et al. 2011), and short-term excursions to deeper waters have also been associated with successful foraging events in shortfin mako sharks (Sepulveda et al. 2004). Blue sharks have also been reported to alter their dive behaviors in response to prey availability (Humphries et al. 2010).

Given the extremely varied tiger shark diet, it is also likely that a wide variety of tactics are used to capture different prey types. Surface-oriented sharks and shark #4 showed very consistent diel dive patterns throughout their tracks (10–29 days), which are similar to those observed in large pelagic predators, such as tunas and billfishes (e.g., Goodyear et al. 2008; Walli et al. 2009; Weng et al. 2009; Hoolihan et al. 2011), and have been linked to foraging on vertically migrating prey. Interestingly, the dive pattern of surface-oriented sharks was the opposite of other large pelagic predators (i.e., sharks made repeated deep dives during the nighttime and were shallower during the daytime). If these dive patterns in tiger sharks are reflective of foraging behavior, these sharks may be targeting a deeper water prey base that is constant both temporally and spatially, such as deep sea cephalopods (Smale and Cliff 1998), which are likely to be more accessible during the night. These consistent dive behaviors were observed in USVI-tagged sharks tagged in different seasons and engaging in different horizontal movements (i.e., sharks that remained around the Puerto Rico-Virgin Islands platform and one that travelled over 1100 km). In addition, this prey source must have high nutritional value to offset the increased energetic costs of repetitive deep diving.

The distribution of tiger sharks across coastal and oceanic waters results in a large overlap with areas used by both coastal and pelagic fisheries. With their propensity to be associated with the substrate in shallow coastal waters (Holland et al. 1999; Nakamura et al. 2011), it is not surprising that tiger sharks constitute 19% of the bycatch in the US Atlantic bottom longline shark fishery (Morgan et al. 2010). In addition, during oceanic forays, the vertical habitat use of tiger sharks suggests they will be exposed to pelagic longline fisheries, which, depending on the target species, fish at depths ranging from 25 m to 400 m (Ward et al. 2009). Indeed, tiger sharks have been recorded in pelagic longline fisheries across their range, but catch rates are usually low (e.g., Polovina and Lau 1993; Beerkircher et al. 2002; Baum and Myers 2004), with some regional exceptions (see Baum and Blanchard 2010).

Although impacted by multiple fisheries, the highly interindividual variable behaviors exhibited by tiger sharks, both vertically and horizontally, may contribute to reducing their exposure to fisheries and thereby catch rate, as only a subset of a tiger shark population will be vulnerable to the fisheries on a local scale. This type of behavior, combined with the traits of relatively high fecundity and high survival rate after capture may explain why tiger shark population declines are not typically as great as other shark species exposed to the same fisheries (Baum et al. 2003; Carlson et al. 2012) and shark control programs (Simpfendorfer 1992; Wetherbee et al. 1994).

The high degree of intraspecific variability observed in tiger shark vertical habitat use makes predicting tiger shark use of the water column and deciphering the forces driving this variable behavior complex. Combining information on horizontal movements and diet with vertical movements may provide insight into the causes of this variability. The observed high intraspecific variability, if a general behavioral feature of tiger sharks, could have wide-reaching evolutionary and ecological consequences (Bolnick et al. 2003, 2011; Wolf and Weissing 2012), especially if differences in vertical behaviors result in dietary differences among individuals. Individual dietary variation can lead to greater connectivity in food webs because predator populations may interact with more prey species and could also lead to lower interaction strengths between predators and their prey because only a subset of the predator population is interacting with each prey species. Both of these features promote stability in food webs (McCann et al. 1998; Gross et al. 2009).

Although behavioral intraspecific variability is common across a wide variety of taxa (Bolnick et al. 2003), few studies have focused on the causes of behavioral intraspecific variability in elasmobranchs (e.g., Sims et al. 2005; Matich et al. 2011; Queiroz et al. 2012). Further, behavioral intraspecific variability likely occurs in many other shark species because individual specialization appears to be common in upper trophic levels (Araújo et al. 2011). Identifying examples of behavioral intraspecific variability in large sharks is important because, as apex predators, large sharks have the potential to play key roles in the dynamics of marine ecosystems (Heithaus et al. 2010). Recognizing behavioral variability is also crucial because it can have conservation implications; management designed around “average” resource use may be of limited value in species with considerable variation (Bolnick et al. 2003). Elucidating the drivers of such high intraspecific behavioral variability in tiger sharks will likely require the combination of multiple techniques, coupling high resolution vertical and horizontal movement data, as well as detailed examination of the trophic ecology and environmental conditions experienced by sharks while their movements are monitored.
